# MTH1 counteracts oncogenic oxidative stress

**DOI:** 10.18632/oncoscience.240

**Published:** 2015-09-12

**Authors:** Dominick G.A. Burton, Priyamvada Rai

**Affiliations:** Rosenstiel Medical Sciences Building, University of Miami Miller School of Medicine, Miami, FL, USA

**Keywords:** MTH1, oncogenic stress, RAS, p53

Oncogenic oxidative stress is an intrinsic part of the transformation process and needs to be mitigated for optimal malignancy in the transformed cancer cells. The source of oncogenic reactive oxygen species (ROS) likely differs depending on the stage of transformation and can be, separately or collectively, the result of hyperactivated oncogenic signaling, inflammation, aberrant mitochondrial metabolism, and instigation of invasion-promoting pathways[1]. In RAS-driven tumor cells, we have found that MTH1, the mammalian Nudix hydrolase that degrades oxidized purine nucleotides, effectively counteracts the resistance to transformation and malignancy from this inherent oxidative stress. Our work was the first to show that MTH1 is required to facilitate the formation, proliferation and tumorigenic capability of RAS-transformed tumor cells [2–4], thereby establishing a critical role for the oxidation state of the nucleotide pool in determining the malignancy of RAS-driven cancer cells. It is plausible that in the context of other tumor-associated oxidative stress, besides RAS activation, MTH1 would perform a similar protective function although this premise has yet to be explicitly tested.

In effect, MTH1 loss compromises the overall robustness of the transformation circuit and enables the negative consequences of oncogenic oxidative stress on tumor formation. Elevated oncogenic ROS can impair the transformation process by promoting genomic DNA damage and anti-tumor processes such as oncogene-induced senescence (OIS) or cell death. Yet ROS-dependent signaling is essential for oncogenic transformation [5]. This duality of ROS drives the acquisition of molecular adaptations that uncouple the tumor-promoting aspects of ROS from their tumor-suppressive consequences.

Therefore, the importance of MTH1 in RAS transformation likely lies in its prevention of oxidative DNA damage and the resulting anti-proliferative consequences in the absence of any ROS scavenging functionality[1]. This characteristic places MTH1 in a unique class of non-oncogenic adaptation – the ability to mitigate the negative influences of ROS on tumor formation without directly altering the ROS levels required for oncogenic signaling. Other molecules in this class of adaptations, deriving from DNA damage repair or non-antioxidant redox-protective pathways, are anticipated to possess a similar uncoupling effect.

Whilst MTH1 may not be able to directly alter ROS levels, its loss can force reductions in oncogenic oxidants by selectively eradicating cells with high levels of RAS oncoprotein and/or ROS-generating downstream pathways that cannot cope with the consequences of MTH1 inhibition due to their elevated oxidant status. Significantly, our recent findings [4] demonstrate that this phenomenon occurs in p53-nonfunctional lung cancer cells that are able to resist MTH1 inhibition-induced genomic DNA damage and continue to proliferate despite MTH1 loss, albeit at a slower rate. It is well known that approximately 50% of all tumors contain p53 mutations or loss that make them refractory to stresses that induce DNA damage. We find that p53 is required for MTH1 inhibition-induced DNA strand breaks and senescence[4]. However even in the absence of functional p53, MTH1 inhibition reduces tumor formation and proliferation rates. This seems to occur through an additional adaptation that involves a gradual decrease in ROS levels, arising ostensibly from a striking reduction in RAS oncoprotein and activated Akt levels in both the MTH1-inhibited cultured cells and xenograft tumors[4].

We observed a similar effect when we introduced a CMV promoter-driven high RASV12-expressing construct into shMTH1-transduced immortalized lung cells[3]. Significantly, the reduction in ROS and in RAS levels was much milder when we instead introduced a low RASV12-expressing construct, which generated lower oncogenic ROS levels relative to the high RASV12 construct[3]. Similarly KRAS-driven cells in a low oxidative stress environment, created through low oxygen culture, no longer responded to MTH1 inhibition by a selective reduction in RAS oncoprotein levels[4]. Conversely, if ROS were maintained at elevated levels under MTH1 inhibition, for instance by the enforced expression of activated Akt, RAS-driven cells suffered a greater proliferative deficit than if they were able to shift to a lower ROS-producing population[4].

**Figure 1:**
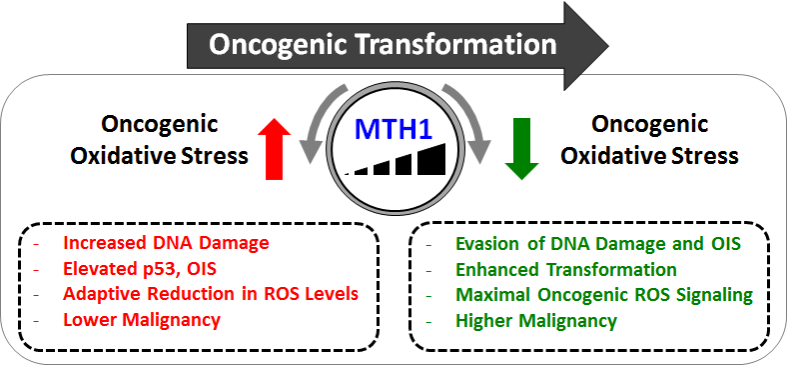
MTH1 functions as a molecular rheostat in oncogenic transformation High MTH1 levels facilitate maximal oncogenic malignancy by enabling evasion of the adverse consequences from transformation-associated oncogenic ROS. Conversely, inhibition of MTH1 allows oncogenic oxidants to limit malignancy. In effect, MTH1 negates the resistance to transformation from oncogenic ROS signaling-associated oxidative stress.

Taken collectively, these results suggest three things: 1) the higher the RAS oncoprotein expression (as seen in advanced tumors), the greater the requirement for MTH1 expression to counteract the related high oncogenic ROS-associated negative effects, 2) the loss of MTH1 leads to a selective decrease in cells with the highest RAS expression (and consequently ROS levels) in a tumor population, and 3) in cells resistant to MTH1 inhibition-induced DNA damage and senescence, the very fact of their continued proliferation necessitates the adaptive response of decreasing RAS-mediated ROS production, at the cost of reduced malignancy. It thus appears that MTH1 could function as a molecular rheostat for modulating oncogene expression at optimal levels, in turn controlling cancer survival and growth in RAS-driven cells (Figure 1).

As an increase in genomic strand breaks was not detected in the p53-nonfunctional RAS-driven cells following MTH1 depletion in our study[4], it begs the question as to what specific stressor(s) the cells are sensing and responding to by shifting to an overall lower ROS-producing population. An understanding of the molecular processes involved is essential for the development of further therapeutic strategies to indirectly target RAS activity or response via MTH1 inhibitors. One such avenue of investigation could involve the understudied interplay between unrepaired genomic 8-oxoG, ROS and RAS activity. Our unpublished results suggest that p53-nonfunctional cells possess low levels of the base excision repair (BER) protein, OGG1. A recent study postulated that OGG1 can activate RAS signaling by acting as a guanine nucleotide exchange factor (GEF) when bound to the excised 8-oxoguanine moiety[6]. Accordingly impaired OGG1-mediated repair of genomic 8-oxoG resulting from MTH1 inhibition could in turn lower RAS activation and associated ROS production. In addition to producing ROS, RAS activity is itself modulated by oxidants such as the superoxide radical[7], suggesting the existence of a ROS-mediated positive feedback loop that sustains RAS activation. Thus the net effect of low OGG1 in the context of MTH1 inhibition may be to further reduce RAS activity and associated ROS levels, leading to a reduction in associated protective effects and the shift to a less malignant cell population.

Regardless of the specific mechanism(s) involved, the ability to indirectly modulate RAS activity/expression by targeting MTH1 is an attractive approach, given the historical difficulties in direct therapeutic targeting of RAS and its downstream effectors. Our approach further suggests that targeting an unavoidable oncogenic vulnerability such as oxidative stress may provide a more efficacious broad-spectrum therapeutic response than targeting oncogenic pathways, which have proven to be pleiotropic and mutable.
